# Iliopsoas Abscess

**DOI:** 10.12669/pjms.37.2.3816

**Published:** 2021

**Authors:** Bader Sobaih, Loay Sobaih, Fahad Al Zamil

**Affiliations:** 1 Bader Sobaih, Department of Pediatrics, College of Medicine, King Saud University, Riyadh, Saudi Arabia; 2 Loay Sobaih, Department of Pediatrics, College of Medicine, King Saud University, Riyadh, Saudi Arabia; 3 Fahad Al Zamil, Department of Pediatrics, College of Medicine, King Saud University, Riyadh, Saudi Arabia

**Keywords:** CT Scan, Iliopsoas abscess, Limping

## Abstract

Iliopsoas abscess (IPA) is uncommon condition in children, diagnosis might be delayed because of nonspecific signs and symptoms. Only few patients have classical clinical triad at presentation in the form of fever, back pain, and inguinal pain at hip flexion. The diagnosis most likely to be reached in the first time by the use of abdominal computed tomography (CT) scan. We present a Saudi child with nonspecific signs and symptoms of fever, flank pain, and limping who was diagnosed as IPA by abdominal ultrasound and CT scan. The case was managed with intravenous antibiotics along with transcutaneous abscess drainage.

## INTRODUCTION

Iliopsoas abscess (IPA) is uncommon in children, management might be delayed as a result of its varied signs and symptoms which present the patient to many specialties. Earlier diagnosis can be made by the use of imaging modalities such as ultrasound, and computed Tomography (CT) scan.^1^ The reported incidence in the United Kingdom is 0.4/100,000.^2^ Ilio-psoas abscess could be primary due to the hematogenous or lymphatic spread of a causative organism, from a distant site or secondary as a result of the direct expansion of a nearby infectious / inflammatory process into the ilio-psoas. We report two cases of Ilio-psoas abscess in Saudi children.

## CASE REPORT

A 7-year-old Saudi boy presented to emergency department with 10 days history of high-grade fever (39-40^º^C) and left sided flank pain. The pain was progressive, stabbing in nature, aggravated by movement and standing, and relieved by sitting. Pain was not radiated, and severity scored was 9/10. He had past history of blister in the anterior abdominal wall which ruptured spontaneously with greenish pus discharge. Also had history of recurrent peri-oral ulcers, recurrent diarrhea without vomiting during the last year. The father had one-year history of abscess in multiple sites; eyes, foot, and thigh. The mother had two abscesses below the left armpit, started two months ago, and resolved spontaneously without seeking medical advice. His sister suffered one large abscess in the chest area which ruptured spontaneously three months back. On examination, patient was febrile (38.8^º^C), with normal pulse rate, heart rate, and blood pressure. Abdomen was soft, no organomegaly. There was swelling over the left flank area measuring 2x3 cm, which was tender and warm with no redness of skin, systemic examinations were normal.

Complete blood count (CBC) showed leukocytosis (14000) with neutrophilia (74%). Erythrocyte sedimentation rate (ESR) was 120, C-reactive protein (CRP) was 160. Renal function, and liver function tests were normal. Blood culture and urine culture showed no growth of any organism. Acid fast bacilli test was negative. Renal ultrasound revealed multilobulated, multiseptated fluid collection posterior to the left kidney measuring about 5 x 2.5 cm with intramuscular component. There was a focal heterogeneous area in the posterior cortex of left kidney in relation to the collection that could represent renal involvement. Kidneys demonstrate normal cortical echogenicity and preserved cortical medullary differentiation, no evident stone or hydronephrosis. Abdomen CT scan showed multi-lobulated, multiseptated fluid collection posterior to left kidney extending to back muscles as described. Ultrasound-guided posterior per-nephric intramuscular collection drainage was done. Approximately 20 ml of frank thick pus was aspirated, which was negative for acid fast bacilli. Culture was negative for fungi but positive for Methicillin-resistant Staphylococcus aureus (MRSA). Patient was accordingly started on Clindamycin.

**Fig.1:**
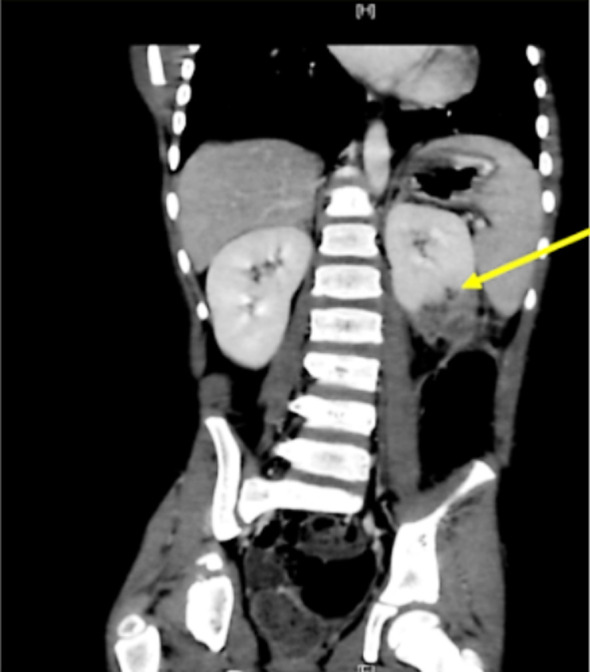
Cortical hypodensity in the posterior cortex reflect extension into renal parenchyma.

## DISCUSSION

Iliopsoas abscess (IPA) is a rare condition that may affect children as well as adults. The psoas muscle (PM) has anatomical relation with kidneys, ureters, pancreas, appendix, cecum, colon, and lumber lymph nodes. Patients may feel pain if any of these organs are compromised. Infection in any nearby organs may spread to the PM through contiguity.^3^

IPA is classified as primary and secondary. Primary IPA is considered when there is no obvious local cause and due to hematogenic spread of infectious from any part of the body. Secondary IPA is mainly caused by spread of infection from adjacent organ. Primary IPA became more prevalent in young patients.^4^ The clinical presentation of IPA is variable and diagnosis often not considered at first. Fever, back pain, and inguinal pain associated with hip flexion, is the classical clinical presentation triad for IPA, but unfortunately, this typical clinical presentation is found in minority of cases which may lead to delayed diagnosis of IPA which will be often first diagnosed by abdominal CT scan.^5^

**Fig.2:**
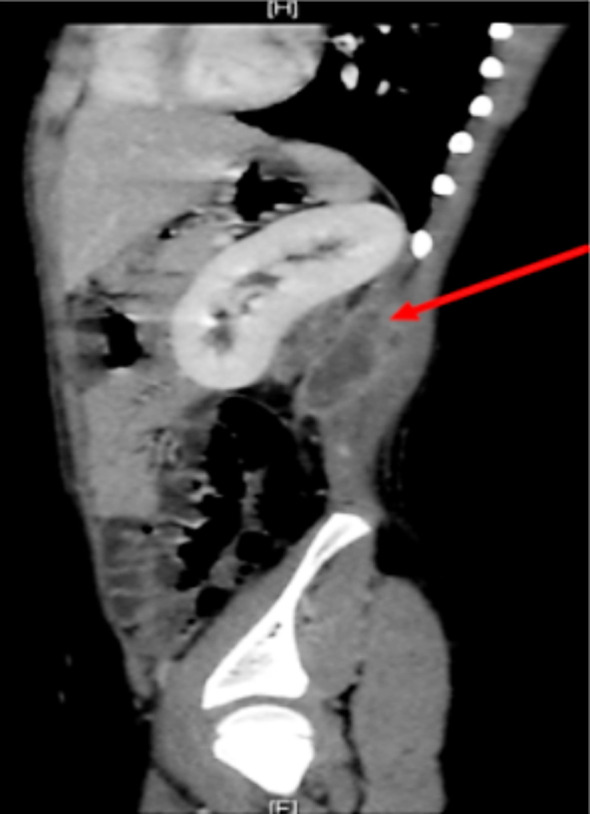
Multiloculated collection in relation to the muscles mainly left iliopsoas.

The case was investigated to find out the causative organism and to elaborate if there is any involvement of adjacent organs. Abdominal ultrasound and CT scan revealed the presence of left kidney abscess. Methicillin-resistant staphylococcus aureus (MRSA) being the underlying causative organism. The reported case presented with typical triad of IPA. The patient presented with flank pain which may explain the presence of left renal abscess, the diagnosis of IPA was first made by abdominal ultrasound and CT scan, patient was treated by appropriate antibiotics in conjunction with drainage of abscess.

## CONCLUSION

IPA is a rare disease in children and should be suspected when a patient presents with fever, lower abdominal pain, and decreased hip movement. Broad spectrum Intravenous antibiotics with coverage of S. aureus and image-guided percutaneous drainage are effective in managing most patients. Open drainage is required if percutaneous drainage fails to completely resolve the abscess and clinical symptoms deteriorate despite antibiotic treatment.

### Authors’ Contribution.

**Bader Sobaih:** Manuscript writing.

**Loay Sobaih:** Literature review.

**Fahad Al Zamil:** Manuscript editing, is responsible for integrity of the study.
